# Local food environment interventions to improve healthy food choice in adults: a systematic review and realist synthesis protocol

**DOI:** 10.1136/bmjopen-2014-007161

**Published:** 2015-05-02

**Authors:** Tarra L Penney, Helen Elizabeth Brown, Eva R Maguire, Isla Kuhn, Pablo Monsivais

**Affiliations:** 1 UKCRC Centre for Diet and Activity Research (CEDAR), MRC Epidemiology Unit, University of Cambridge School of Clinical Medicine, Cambridge, UK; 2 Medical Library, University of Cambridge, School of Clinical Medicine, Cambridge, UK

**Keywords:** PREVENTIVE MEDICINE, PUBLIC HEALTH, QUALITATIVE RESEARCH

## Abstract

**Introduction:**

Local food environments have been linked with dietary intake and obesity in adults. However, overall evidence remains mixed with calls for increased theoretical and conceptual clarity related to how availability of neighbourhood food outlets, and within-outlet food options, influence food purchasing and consumption. The purpose of this work is to develop a programme theory of food availability, supported by empirical evidence from a range of local food environment interventions.

**Methods and analysis:**

A systematic search of the literature will be followed by duplicate screening and quality assessment (using the Effective Public Health Practice Project tool). Realist synthesis will then be conducted according to the Realist And Meta-narrative Evidence Syntheses: Evolving Standards (RAMESES) publication standards, including transparent appraisal, synthesis and drawing conclusions via consensus.

**Dissemination:**

The final synthesis will propose an evidence-based programme theory of food availability, including evidence mapping to demonstrate contextual factors, pathways of influence and potential mechanisms. With the paucity of empirically supported programme theories used in current local food environment interventions to improve food availability, this synthesis may be used to understand how and why interventions work, and thus inform the development of theory-driven, evidence-based interventions to improve healthy food choice and future empirical work.

**Trial registration number:**

PROSPERO CRD42014009808.

## Background

A clear link has been found between diet and the prevention of chronic diseases including cardiovascular disease,^1–3^ diabetes,^4^ certain types of cancer^5^ and conditions such as overweight and obesity.^6^ ^7^ As a result, governments are seeking actionable evidence to improve diet in whole populations, propelling a shift in focus from individual-level determinants to policies and environments that elicit, maintain and distribute risk factors across the population.^8–13^ Subsequently, factors beyond the individual have been posited within different socioecological, or multilevelled frameworks that attempt to account for multiple influences including sociodemographics, perceptions of food environments, community food environments (ie, availability and accessibility of outlets), consumer food environments (ie, availability of foods, prices and promotions) and policy.^14–23^ However, placing diet behaviour within the broader socioecological context has brought forward several conceptual and methodological challenges.^24^ ^25^ Principle among these has been the need for socioecological theories that can *simultaneously* account for factors at the intrapersonal, interpersonal, institutional, community and public policy levels.^26^ ^27^ While taking important steps forward, these socioecological frameworks are often developed at a level of abstraction that make determining important points of intervention challenging, a common occurrence that has been criticised more broadly in public health research.^18^ ^28^ This suggests that while socioecological frameworks may expand our view of potential determinants of healthy eating behaviour, they often do so without the needed specificity to improve our understanding, or guide intervention strategy development.^29^ Thus, a major challenge for those working in areas of public health is to improve specificity of socioecological theories needed to better understand both individual and environmental determinants of diet and obesity.^30^

### The local food environment, diet and obesity

As part of the socioecological system, local food environment factors such as food availability (ie, adequacy of the supply of healthy food, examples include the presence of certain types of food outlets, and the number or ‘mix’ of outlets to purchase food),^31^ have been associated with less healthy diets and increased body weight in adults.^32^ ^33^ However, overall evidence remains mixed,^34^ ^35^ with several calls in the literature for increased conceptual and theoretical clarity on *how* availability of food outlets, and within-outlet food options influence diet behaviour.^22^ ^36^ Although several systematic reviews have been conducted on the topic of food environment and diet behaviour or obesity,^31^ ^37–40^ their conclusions regarding the effectiveness of various strategies are not definitive. One reason for this gap may be the review synthesis method used to examine a range of heterogeneous food environment interventions. The focus of systematic reviews of food environment literature to date has been to summarise observational studies regarding neighbourhood food environments, diet and obesity,^31^ ^41^ ^42^ to identify food environment interventions and their effectiveness to improve diet or reduce obesity (ie, small outlet interventions, prepared food outlet interventions),^37^ ^40^ to synthesise evidence describing different potential strategies (ie, change in food outlet offerings),^38^ ^39^ or to focus on the methods used in food exposure and outcome assessment (ie, food purchasing or diet quality).^31^ Additionally, these reviews do not conceptually differentiate between different food environments, specifically studies focused on intervention strategies that target issues of availability and/or accessibility at both the community and/or consumer level (ie, location of a food outlet with respect to where people live vs the food sold by an outlet) and other exclusively consumer–environment interventions. Typically, the latter intervention strategies do not necessarily target *what* food is sold but rather *how* food is sold including the use of promotions, placement or point-of-purchase information. However, both types of intervention strategies are often reviewed together, or in some cases the intervention itself makes use of these strategies simultaneously. For example, a premade food outlet intervention might introduce point-of-purchase information and additional healthy offerings together, making the determination of relative contribution of different strategies, their potential interactions, or even the hypothesised mechanisms of influence, challenging to tease apart. Furthermore, there are direct policy implications for the independent investigation of food availability as a necessary condition for healthy diets, and to support reductions in levels of obesity. Currently, there is global discourse^43^ ^44^ related to possible planning laws to regulate the growth of fast food and unsupportive built environments in some countries,^45^ ^46^ where a clear understanding of the influence of neighbourhood and outlet food availability, diet and obesity is needed.

While it is necessary to summarise intervention strategies, examine effectiveness and critically evaluate methods, the complex nature of food environment interventions (and their direct relevance to healthy public policy) may require examining the current evidence base from a new perspective, employing a research synthesis capable of dealing with greater complexity and a focus on *how, for whom and under what conditions* food availability interventions exert their hypothesised effects. Therefore, the purpose of this work is to conduct a review based on a systematic search of food environment interventions and a realist synthesis of all intervention evidence. Local food environment interventions will include only those that sought to improve food availability at the neighbourhood (ie, the introduction of a food outlet) or outlet (ie, the introduction of foods) levels. Specifically, the primary objective will be to examine evidence to help develop a theory of food availability to answer the following questions:

- How does a change in food availability influence diet?
- For whom does a change in food availability influence diet?
- Under what circumstances does a change in food availability influence diet?

## Methods

The protocol is registered with the International Prospective Register for Systematic Reviews (PROSPERO) CRD42014009808. Ethical approval was not required for the start of this study.

Food environment interventions can be described as complex interventions that aim to modify various levels of influence on dietary behaviours. Therefore, the methodology employed in this systematic review is realist synthesis, a theory-driven approach that holds its foundations in realist philosophy of science.^47^ Additionally, the approach inherently provides focus on understanding causation, and how causal mechanisms are shaped and/or constrained by a broader multilevelled context. The following section will outline the review procedures as recommended by the Realist And Meta-narrative evidence Syntheses: Evolving Standards (RAMESES) publication standards.^48^

### Scoping, identification and screening of articles

**Figure 1:**
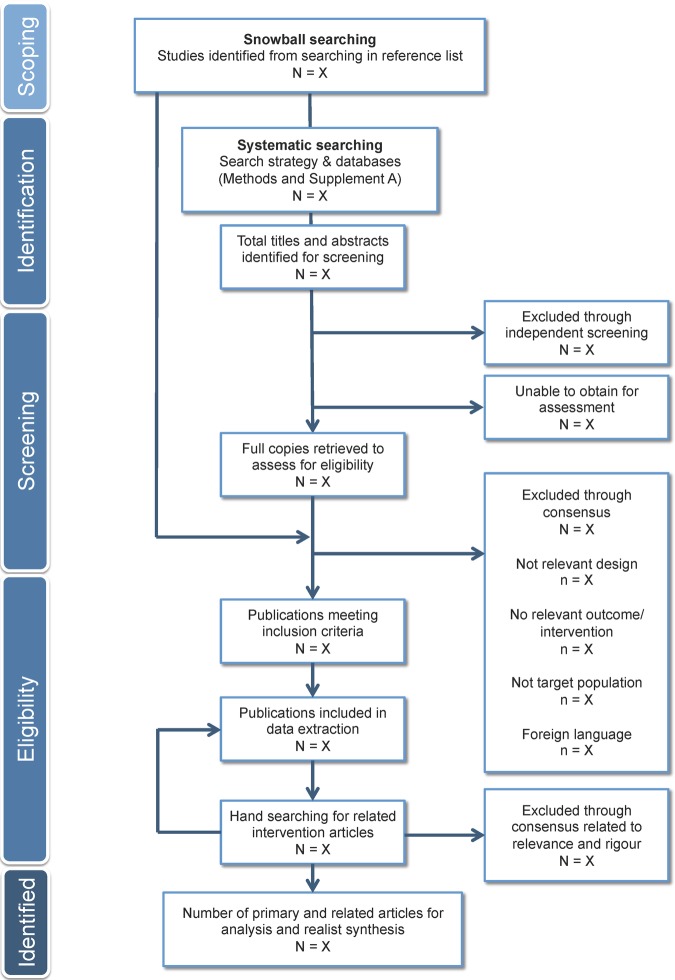
Flow diagram for search process and study selection.

Given the complexity of the food environment intervention literature, scoping for this work will help to further clarify the conceptualisation of food environment interventions that will be the focus of the review. This will be achieved through a snowball searching of five review articles focusing on interventions in a range of settings that have been identified.^31^ ^37–40^ These interventions will also be used to develop and test the data extraction and quality assessment tools for the full systematic review prior to the systematic search (figure 1).

The subsequent systematic search will be conducted by a medical librarian (IK), and the resulting literature will then be de-duplicated and exported to Endnote X7.2. The following databases will be searched for articles published up to and including July 2014, with no limit on earliest year of publication; MEDLINE (Ovid SP), EMBASE (Ovid SP), PsychINFO (Ovid SP), EconLit (EBSCO), Applied Social Sciences Index and Abstracts (CSA Illumina) and Cochrane Database of Systematic Reviews (Wiley Online Library). The search strategy will be common across databases (full strategy available in online supplement A).

A particular tenet of the realist synthesis approach is the inclusion of a range of evidence sources, and an emphasis on iterative search processes. Therefore, in addition to the screening for peer-reviewed outcome evaluations for interventions, hand searching will be conducted for each intervention selected to identify (1) peer-review publications that were secondary to the outcome evaluation including process evaluations and (2) grey literature in the form of websites, final project reports or short articles discussing the context of the intervention being conducted.

All retrieved titles and abstracts will be screened by the primary author (TLP), and relevant items duplicate screened by another author (ERM). Criteria for screening will be refined if necessary, and any discrepancy in inclusion or exclusion will be resolved through a consensus discussion between authors (TLP, ERM, HEB and PM). Full-text versions of selected articles from both the systematic and the hand search will then be obtained, and inclusion and exclusion criteria assessed (following a similar procedure as for titles and abstracts; duplicate screening and consensus discussion between TLP and ERM with disputes settled by a third author (HEB or PM)).

### Eligibility and quality assessment of articles

Primary intervention studies will be screened for inclusion based on (1) seeking to improve diet through a change in the availability of outlets (ie, the opening of a new outlet) or the availability of foods in outlets (ie, new food items in an outlet); (2) including food outlets that do not have restriction of use including convenience outlets, small food outlets, grocery outlets, takeaway outlets or full service sit-down restaurants; (3) including adults aged ≥18 years at baseline; (4) reporting on results from a measure of diet alone (ie, diet quality or food purchasing), or diet and a measure of obesity (ie, body mass index) and (5) having been published in a peer-reviewed journal or grey literature sources (ie, websites or programme reports) up to and including July 2014. Study designs may include randomised controlled trials, comparison trials and/or quasiexperimental studies. Interventions that *do not* report a measure of diet as the primary measure, but include a measure of body mass index alone will be excluded. Studies of adults that represent special populations (including pregnant woman or clinical populations) will also be excluded. Any interventions that examine aspects of the food environment in the absence of a change in food provision within a neighbourhood or an outlet will be excluded. These exclusion criteria are to ensure results are congruent with the review objectives. Results of the duplicate screening will be reported using figure 1.

Quality assessment will be conducted by the lead author (TLP) using the Effective Public Health Practice Project (EPHPP) Quality Assessment Tool for Quantitative Studies; this will be duplicated by an additional author (ERM), and inter-rater reliability reported as a percentage of items without initial consensus. The EPHPP tool rates studies as ‘strong’, ‘moderate’ or ‘weak’ using six scales (selection bias, study design, confounders, blinding, data collection methods, and withdrawals and drop-outs). Studies are then rated to give an aggregate overall score of ‘strong’, ‘moderate’ or ‘weak’ (‘strong’ if no ‘weak’ individual-scale ratings are designated, ‘moderate’ if 1, and ‘weak’ if 2 or more). The tool has been recommended for use in assessing public health interventions based on acceptable content and construct validity,^49^ and the results will be reported for each of the primary intervention studies. Quality assessment of additional peer-reviewed process evaluation studies, or grey literature articles, will not be undertaken, as these studies will be used primarily as contextual details in the realist synthesis.

Summary data regarding study participants, intervention setting and characteristics, and outcomes, will be extracted by the primary author (TLP), and checked for accuracy by another author (ERM). Discrepancies will be resolved through consensus discussion. Descriptive data on all primary intervention studies will be reported (table 1).

**Table 1:** Summary table for extracted data from included studies

Study (first author, year and country)	Design (study design, randomisation, control)	Participants (baseline and follow-up)	Diet measure (time period, measurement type)	Intervention (name, strategy, duration, theory)	Outcome (diet change)	EPHPP score (global)
001	**…**	**…**	**…**	**…**	**…**	**…**
002	**…**	**…**	**…**	**…**	**…**	**…**

EPHPP, Effective Public Health Practice Project.

### Analysis and realist synthesis process

As per realist synthesis procedural recommendations^48^ and methods employed in other research on health behaviour,^50^ ^51^ a phased but iterative approach will be taken. First, an ‘initial’ theory will be developed, utilising knowledge from the study team and selected content experts where needed. This programme theory will describe the context and mechanisms necessary to trigger a specified outcome (namely, diet behaviour for adults).

Next, the inclusion of studies to inform further programme theory development through included studies will be guided by the principles of ‘relevance’ (ie, whether the data can contribute to theory building and/or testing) and ‘rigour’ (ie, whether the method used to generate the data is credible and trustworthy).^48^ The data examined will be coded within each primary (ie, outcome evaluation study) and secondary (ie, process evaluation or grey literature) intervention study using ATLAS.ti qualitative analysis software (first by one author (TLP), and then reviewed by a second author (HEB)). Coding will be guided by the initial programme theory and the review questions of how food environment interventions work, for whom and under what conditions, with the purpose of exploring data on context, mechanisms and outcome configurations, patterns and stated programme theories for the included interventions. Further relevant evidence will be sought via hand searching, if needed, and fed into the overall search and analysis procedure.

Data synthesis will then involve interpreting and mapping the results against the initial programme theory to identify areas of strength, and areas that require further research. The programme theory will then be refined to reflect mechanisms that are supported by evidence. If appropriate, existing substantive theory to corroborate stages of the programme theory will be sought, repeating the above process as required, in order to iteratively test the evolving programme theory and refinement of the theoretically based explanations using included studies as data sources.

## Discussion

The role of the food environment in healthy eating at the population level is an important area of investigation, with several research syntheses seeking to better understand the complex relationship. Although we are still in need of clarity regarding the mixed results related to the effect of food environments on diet, policies to improve diet by altering food environments are currently being implemented and discussed.^43–45^ Often, these actions occur without a clear empirically-derived theoretical basis for how, for whom and under what conditions food environments exert their influence. Therefore, the proposed synthesis will offer an evidence-based programme theory of food availability for diet behaviour, including evidence mapping to demonstrate contextual factors, pathways of influence, and potential mechanisms. With the paucity of empirically supported programme theories used in current local food environment interventions to improve food availability, this synthesis may be used to understand how and why interventions work, and thus inform the development of theory-driven, evidence-based interventions to improve healthy food choice and future empirical work.

### Potential limitations

This work has some potential limitations. The purpose of this realist synthesis is to focus on contextual factors and develop a theory of food availability and diet; however, it will do so without directly assessing intervention effectiveness. Further, in order to provide the most comprehensive understanding of how the included food environment interventions work, this review will be more inclusive of studies than traditional systematic reviews, giving rise to questions of the quality of included studies. Although studies of low quality according to our tool will not be excluded, the quality score will help us during analysis, synthesis and theory-testing stages.

### Dissemination

The results of this study will be disseminated to academic and non-academic audiences through peer-reviewed publications, conferences, formal presentations to policymakers and practitioners, and in formal stakeholder meetings.
